# Pylorus Preserving Pancreatoduodenectomy Versus Whipple Procedure for Adenocarcinoma of The Head of The Pancreas

**DOI:** 10.1155/1989/94691

**Published:** 1989

**Authors:** Lino Belli, Franco Riolo, Federico Romani, Fabio Baticci, Ornella Rossetti, Maurizio Puttini

**Affiliations:** Department of Surgery Pizzamiglio II Niguarda Hosptial Milan Italy

## Abstract

Thirty eight patients underwent pancreatoduodenectomy for histologically confirmed adenocarcinoma of the head of the pancreas. Twenty one underwent a pylorus preserving pancreatoduodenectomy and seventeen the classical Whipple procedure. We undertook this retrospective analysis to compare longterm survival following the two different surgical procedures.

Patients in the two groups were comparable for preoperative laboratory data, age and pathological staging. Minor and major morbidity was not different between the two groups (33.3% and 35.2% respectively). In the pylorus preservation group a delayed resumption of full oral diet and a consequent prolonged hospital stay has been noted (;21.3 days vs 15.4 days, p<0.05). Mean survival was 21 months in the pylorus preservation group and 17 in the Whipple group. No statistical difference was observed between the two survival curves. According to these data the pylorus preserving pancreatoduodenectomy represents a reasonable option for adenocarcinoma of the head of the pancreas.


*HPB Surgery* 1989, Vol. 1, pp. 195–200
Reprints available directly from the publisher
Photocopying permitted by license only

© 1989 Harwood Academic Publishers GmbH
Printed in Great Britain

### PYLORUS PRESERVING PANCREATODUODENECTOMY VERSUS WHIPPLE PROCEDURE FOR ADENOCARCINOMA OF THE HEAD OF THE PANCREAS

LINO BELLI, FRANCO RIOLO, FEDERICO ROMANI*, FABIO BATICCI, ORNELLA ROSSETTI and MAURIZIO PUTTINI

*From Department of Surgery Pizzamiglio II, Niguarda Hosptial, Milan, Italy*

*(Received 28 July, 1988)*

Thirty eight patients underwent pancreatoduodenectomy for histologically confirmed adenocarcinoma of the head of the pancreas. Twenty one underwent a pylorus preserving pancreatoduodenectomy and seventeen the classical Whipple procedure. We undertook this retrospective analysis to compare longterm survival following the two different surgical procedures.

Patients in the two groups were comparable for preoperative laboratory data, age and pathological staging. Minor and major morbidity was not different between the two groups (33.3% and 35.2% respectively). In the pylorus preservation group a delayed resumption of full oral diet and a consequent prolonged hospital stay has been noted (21.3 days vs 15.4 days, $p<0.05$). Mean survival was 21 months in the pylorus preservation group and 17 in the Whipple group. No statistical difference was observed between the two survival curves. According to these data the pylorus preserving pancreatoduodenectomy represents a reasonable option for adenocarcinoma of the head of the pancreas.

KEY WORDS: Adenocarcinoma of the pancreas, pylorus preserving pancreatoduodenectomy, whipple operation

### INTRODUCTION

Since 1978, when reintroduced by Traverso and Longmire [1], pancreatoduodenectomy with pylorus preservation has gained a growing popularity probably because the reconstruction is simpler and the belief that leaving an intact stomach and pylorus would prevent postgastrectomy problems.

Although initially proposed for benign disease several patients have undergone this procedure for neoplastic lesions [2], despite the concern that the reduction of the resected area, compared with the classical Whipple operation, could adversely influence longterm survival. Previously only two series [3,4] addressed this problem and both reported similar survival following pylorus preservation and Whipple procedures, but in one [3] the majority of patients had carcinoma of the ampullary region, so that quantitative data are still not conclusive.

With the aim of further examining this issue we retrospectively studied 38 patients

* Address correspondence to: Federico Romani M.D., Department of Surgery Pizzamiglio II, Niguarda Hospital, Milan, Italy with resectable adenocarcinoma of the head of the pancreas to compare the two procedures.

### METHODS

From 1973 to 1986 38 patients had a partial duodenopancreatectomy for a histologically confirmed adenocarcinoma of the head of the pancreas.

Twentyone underwent the pylorus preservation procedure and seventeen the Whipple procedure. Mean age was 56 years (range 42–75), 22 were men and 16 women. Mean age, male to female ratio and preoperative laboratory data (76% of patients were jaundiced, 28% were malnourished with albumin level less than 3 gm./ml, 21% were hyperglycaemic) were similar for both groups.

In all cases an initial throrough exploration of the abdomen excluded the presence of secondary deposits in the liver, omentum, mesentery or pelvic shelf as well as adhesion of the mass to the aorta or inferior vena cava. Invasion of vascular structures such as the portal vein and superior mesenteric vessels was also a contra-indication to resection.

The whole of the gland was then examined in order to exclude gross involvement beyond the planned line of transection. It is not our practice to routinely performed frozen sections of the pancreas.

The pylorus preservation operation has been performed by dividing the duodemun 1–2 cm distal to the pylorus and leaving intact the right gastric artery.

A duodenojejunal anastomosis was performed end to side in 15 cases and end to end in 6 cases in which the pancreatic remnant was not anastomized to the jejunum. These cases and 3 additional patients in the Whipple group were managed by intraductal injection of solidifying agent (Ethiblock, Ethicon).

Pathological staging was carried out according to the Fortner classification [5]: in stage I tumor was confined to the pancreas, in stage II the regional nodes were involved while in stage III metastis beyond regional nodes were present. 7 patients with stage I and 10 with stage II were found in the Whipple group while 9 patients and 12 respectively in the pylorus preservation group. Neither the status of the resected margins not the tumour size were significantly different between the two groups (Table 1).

Follow up data for survival curve estimation could be obtained in 15/17 patients in the Whipple group and in 18/21 in the pylorous preservation group (two patients, one in each group, were lost to follow up).

Results are presented as mean ± S.D.

The student t test was used for statistical comparison.

### RESULTS

Three deaths related to resection occured in the study group of 38 patients accounting for a 7.8% mortality rate. One patient in the Whipple group (5.7%) died on the 78th postoperative as a result of a pancreatic fistula. Two patients in the pylorus preservation group (8.5%) died: one on the 10th postoperative day from sepsis and multiorgan failure and the other in the 6th month after surgery from intraabdominal haemorrhage secondary to a pancreatic fistula.

An analysis of the complications is shown in Table 2. A major postoperative complication developed in 13 of 38 patients for an overall rate of 34.2%. Among the 21 patients in the pylorus preservation group a significant complication occured in 7 (33%) vs 6 of 17 (35.2%) patients in the Whipple group (p: n.s.).

Pancreatic fistula was the most frequent complication; we considered as minor a fistula (output less than 60 cc/day) which healed spontaneously without requiring surgery or somatostatin infusion and without interfering with oral feeding, all others were termed major fistulae.

**Table 1** Pathological staging of 38 patients with adenocarcinoma of the head of the pancreas.

| | *Pylorus preservation (n°21)* | | *Whipple (n°17)* | |
|---|---|---|---|---|
| | *pt* | % | *pt* | % |
| **Stageing** | | | | |
| Stage I | 9 | (42.8) | 7 | (41.1)* |
| Stage II | 12 | (57.1) | 10 | (58.8)* |
| **Margins of resection clear** | 16 | (76.1) | 15 | (88.2)* |
| **Tumor size (cm)**** | 3.9 ±0.4 | | 4.0 ± 0.3* | |

* p: n.s.
** Mean ± S.D.

Overall, pancreatic fistulae occurred in 9 out 38 patients (23.6%). Seven closed spontaneously but the remaining two patients died and the cause of death was related to the fistula, mortality rate 5.2%.

Considering this problem in relation to the management of the pancreatic remnant we found that in patients managed with intraductal injection of a solidifying agent a fistula developed more frequently (4/9 vs 5/29) but all were minor fistulas. On the contrary a minor fistula was observed only in 1 out 5 cases in the group of patients who had the pancreatojejunostomy and as described above two patients died.

A temporary choledochojejunal fistula developed in two patients (5.2%) (one in each group).

**Table 2** List of major complications.

| | *Pylorus preservation (n°21)* | | *Whipple (n°17)* | |
|---|---|---|---|---|
| | *pt* | % | *pt* | % |
| PANCREATIC FISTULA | 6 | (28.5) | 3 | (17.6) |
| BILIARY FISTULA | 1 | (4.7) | 1 | (5.8) |
| ENTERIC FISTULA | | – | 1 | (5.8) |
| WOUND INFECTION | 1 | (4.7) | 1 | (5.8) |
| TOTAL | 7 | (33.3) | 6 | (35.2) |

Nasogastric decompression was routinely maintained after the Whipple operation for 6–7 days and thereafter oral diet permitted. A delayed resumption of oral food was necessary in nearly two thirds of patients after the pylorus preservation procedure and nasogastric decompression had to be prolonged for a mean of 11.4

days. Metaclopramide administration provided to be of no use in improving gastric emptying. The longer period required to achieve full diet in the pylorus preservation group caused a longer postoperative hospital stay: 21.3 days vs 15.4 days in the Whipple group ($p<0.05$).

During follow up endoscopy was not performed on a routine basis but only after the development of upper abdominal discomfort. Twelve patients (9 in the Whipple group) had endoscopy at different intervals after surgery: one case of anastomotic ulcer and two cases of gastritis were found after Whipple procedure, while no lesion was seen in the pylorus preservation group.

Nutritional status was studied in 13 patients (7 in the pylorus preservation group and 6 in the Whipple group) 6 and 12 months after resection. All the parameters considered were better after the pylorus preservation procedure: mean plasma albumin level was $4.2 \pm 0.4$ g/dL vs $3.4 \pm 0.5$ d/dL (p: $<0.05$), transferrin was $252 \pm 84$ microg/dL vs $210 \pm 62$ (p: n.s.), body weight increase (percent of body weight at discharge) was $+10\% \pm 5$ vs $+4\% \pm 2$ (p:$<0.05$).

Survival curves are presented in Figure 1. At two years survival was 31% in the pylorus preservation group and 20% in the Whipple group (p: n.s.) and mean survival was 21 months and 17 months respectively (p: n.s.). In all cases the cause of death was neoplastic recurrence.

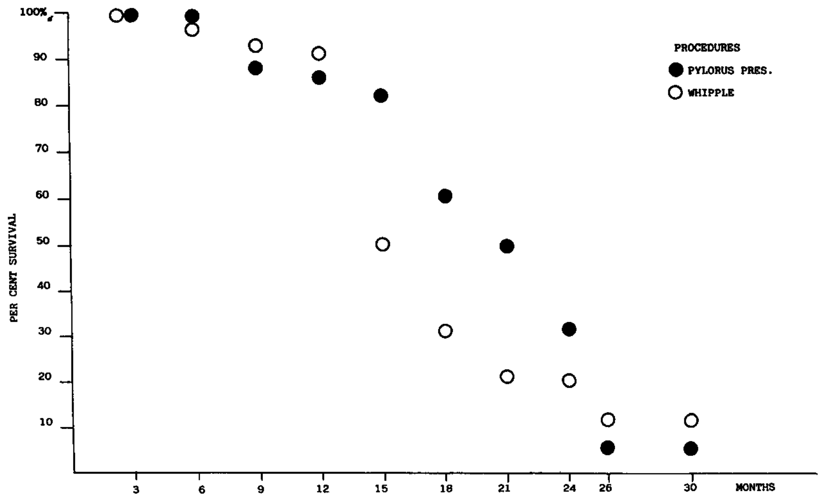

**Figure 1.** Survival curves of patients with adenocarcinoma of the head of the pancreas who underwent a pylorus preservating pancreatoduodenectomy or a Whipple procedure.

### DISCUSSION

In two comparable groups of patients with adenocarcinoma of the head of the pancreas 30 days mortality rate was 4.7% and 0% respectively for pylorus preservation procedure and the classical Whipple procedure. Pancreatic fistula was the most common complication following pancreatoduodenectomy occurring in 23.6% of these patients with a related mortality rate of 5.2%.

Recently in our patients who required pancreatic resection we have prefered to avoid pancreatojejunostomy when there is a soft and friable pancreas with a narrow pancreatic duct. We have managing these pancreatic remnants with injection of a solidifying agents into the duct. This approach did not reduce the rate of fistula formation, but provided less problems in treatment, less discomfort for the patients and no deaths.

A possible criticism of the pylorus preservation pancreatoduodenectomy for carcinoma of the head of the pancreas is that the resected area is reduced and therefore survival may be compromised. Our study indicates that patient survival after pylorus preserving procedure is similar to that after the Whipple procedure, and compares favourably with other reported series of Whipple procedures for pancreatic adenocarcinoma [6, 7, 8]. This observation should not be considered surprising. Histological examination of intraoperative specimen provided by regional pancreatectomies [9] and autopsy studies of patients with early stage of the disease [10] clearly show that adenocarcinoma of the head of the pancreas tend to metastasize early to groups of nodes distant from the pancreas (paraaortic root of the mesentery) usually not removed during the Whipple operation. Furthermore the lymph nodes along the lesser and greater curvature of the stomach were always found to be uninvolved in the series of 33 patients reported by Cubilla [9], as in our series of Whipple operations. Only one out of 148 nodes examined in an autopsy study was found to have evidence of metastasis [10].

A possible concern may be raised by the fact that a clear pancreatic margin of resection could not be obtained in nearly 19% of our patients. In our opinion however this probably has limited clinical influence: survival rates are not worse than in other series, and furthermore in our experience as well as others total pancreatectomy does not improve long term survival [11].

Data from this and our previous studies [3, 4] make pylorus preservation pancreatoduodenectomy a reasonable option for patients with malignancy of the head of the pancreas.

In our experience preservation of the pylorus has a number of advantages over gastric resection. Reconstruction is technically simpler with reduced operating time. Anastomotic ulceration was never observed in the present series and it has been reported as a rare complication even in long term follow up [12]. Finally in agreement with other observations [12] digestive mechanism are well preserved. Thirteen patients were evaluated 6 and 12 months after resection and nutritional status was better in the pylorus preservation group.

The only adverse effect of this procedure was a significant delay in resumption of oral diet. This problem, already reported in the literature [12–14] was observed in the majority of our patients and accounted for the longer period in hospital. In all patients, however, resumption of full diet was eventually achieved. In our opinion some additional days in hospital are a fair price for a better long term nutritional status and quality of life.

In conclusion we suggest, as preferable the pylorus preservation pancreatoduodenectomy for adenocarcinoma of the head of the pancreas because of its advantages over the classical Whipple operation without the risk of a compromized survival.

*Acknowledgement*

We wish to thank Miss Suzy Pizzocaro for kindness in graphic and manuscript preparation.

*References*

1. Traverso, L.W., Longmire, W.P. Jr. (1978). Preservation of the pylorus in pancreaticoduodenectomy. Surg. Gynecol. Obstet. 146: 959–962.
2. Itani, K.M.F., Coleman, E.R., Meyers, W.C., Akwari, O.E. (1986). A clinical and physiological appraisal. Ann. Surg. 204: 655–664.
3. Newman, K.D., Braasch, J.W., Rossi, R.L., O'Campo Gonzales S. (1983). Pyloric and gastric preservation with pancreatoduodenectomy. Am. J. Surg. 145: 152–156.
4. Grace, P.A., Pitt, H.A., Longmire, W.P. (1986). Pancreatoduodenectomy with pylorus preservation for adenocarcinoma of the head of the pancreas. Br. J. Surg. 73: 647–650.
5. Fortner, J.G. (1984). Regional pancreatectomy for cancer of the pancreas, ampulla or other related sites. Ann. Surg. 199: 418–425.
6. Longmire, W.P., Traverso, L.W. (1981). The Whipple procedure and other standard operation approaches to pancreatic cancer. Cancer 47: 1706–1711.
7. Cohen, J.R., Kuchta, N., Geller, N., Shires, T.G., Dineen, P. (1982). Pancreaticoduodenectomy. A 40-year experience. Ann. Surg. 195: 608–617.
8. Edis, A.J., Kiernan, P.D., Taylor, W.F. (1980). Attempted curative resection of ductal carcinoma of the pancreas: review of Mayo Clinic experience 1951–1975. Mayo Clin. Proc. 55: 531–536.
9. Cubilla, A.L., Fortner, J., Fitzgerald, P.J. (1978). Lymph node involvement in carcinoma of the head of the pancreas area. Cancer 41: 880–887.
10. Nagai, H., Kuroda, A., Yasuhiko, M. (1986). Lymphatic and local spread of T and T pancreatic cancer: A study of autopsy material. Ann. Surg 204: 65–71.
11. Van Heerden, J.A. (1984). Pancreatic resection for carcinoma of pancreas. Whipple versus total pancreatectomy – An institutional perspective. World J. Surg. 8: 880–888.
12. Braasch, J.W., Deziel, D.J., Rossi, R.L., Watkins, E., Winter, P. (1986). Pyloric and gastric preserving pancreatic resection. Experience with 87 patients. Ann. Surg. 204: 411–18.
13. Warshaw, A.L., Torchiana, D. (1985). Delayed gastric emphying after pylorus preserving pancreaticoduodenectomy. Surg. Gynecol. Obstet. 160: 1–4.
14. Mosca, F., Giulianotti, P.C., Arganini, M. (1984). Pancreaticoduodenectomy with pylorus preservation. Ital. J. Surg. Sci. 14: 313–320.

*Accepted by S. Bengmark 17 August 1988.*

